# Characteristics of sensorineural hearing loss in tubotympanic chronic otitis media

**DOI:** 10.5339/qmj.2025.96

**Published:** 2025-11-29

**Authors:** Shaimaa Yashar Saadaldeen, Mohammed Radef Dawood, Abdulhussein Kareem Jwery

**Affiliations:** Faculty of Medicine, University of Mustansiriyah, Baghdad, Iraq *Email: shaimaa@uomustansiriyah.edu.iq

**Keywords:** Conductive hearing loss, chronic suppurative otitis media, Iraq, sensorineural hearing loss, tympanic membrane perforation

## Abstract

**Background:**

Chronic suppurative otitis media (CSOM) is a persistent middle ear infection. The tubotympanic type, which is the predominant form, mainly causes conductive hearing loss (CHL), but may result in sensorineural hearing loss (SNHL). This study aims to investigate the frequency and audiological profile of SNHL in patients with tubotympanic CSOM based on a single-center patient population.

**Methods:**

A cross-sectional descriptive study was conducted at a tertiary otolaryngology and audiology unit, from September 2024 to January 2025. Sixty patients with unilateral tubotympanic CSOM were included, with the opposite ear serving as a control. Ethical approval and informed consent were obtained from all participants. Audiological assessments included pure-tone audiometry (PTA), tympanometry, and tuning fork tests. Patients with pre-existing SNHL, prior ear surgery, noise exposure, or other confounders were excluded. Data were analyzed using SPSS v. 28, applying non-parametric tests, with statistical significance set at P ≤ 0.05.

**Results:**

A total of 60 patients were recruited. Twenty-five percent of patients had a disease duration of less than 5 years, 48.3% had a disease duration of 5 to 10 years, and 26.7% had a disease duration of more than 10 years. Presenting symptoms were hearing loss in 86.7%, otorrhea in 45.0%, tinnitus in 36.7%, and dizziness in 13.3%. Fifty-one patients (85%) showed CHL, while nine patients (15%) exhibited mixed hearing loss. SNHL was significantly associated with longer disease duration (P = 0.018) and larger tympanic membrane (TM) perforation (P = 0.036). Bone conduction thresholds were significantly elevated in the diseased ear (P < 0.001), with higher frequencies affected more than speech frequencies.

**Conclusion:**

Tubotympanic CSOM is significantly associated with the development of SNHL, particularly in patients with prolonged disease duration and larger TM perforations. Periodic audiological assessments, proper management, and early intervention are recommended to prevent cochlear damage and permanent hearing loss.

## 1. INTRODUCTION

Chronic suppurative otitis media (CSOM) is a persistent inflammation of the middle ear and mastoid lasting over 12 weeks, characterized by tympanic membrane (TM) perforation and recurrent ear discharge. It is classified into tubotympanic (mucosal) and atticoantral (squamosal) types, with the tubotympanic variety being more prevalent.^1^ CSOM remains a major public health concern, particularly in low-income regions, with poor hygiene and limited healthcare access contributing to its prevalence.^2^ It affects approximately 31 million people annually, with the highest burden in South Asia, Africa, and indigenous populations.^3^ Prevalence varies, exceeding 7% in some Middle Eastern countries (e.g., Yemen, Iran) but remaining below 1% in developed nations, except in high-risk groups.^4,5^ Risk factors include socioeconomic differences as income, education level, and living conditions, that often influence access to healthcare and overall quality of life, recurrent respiratory infections.^6,7^ Hearing loss in CSOM can be conductive, sensorineural, or mixed.^8^ Conductive hearing loss (CHL) results from TM perforation and ossicular damage.^9^ Sensorineural hearing loss (SNHL) can develop due to bacterial toxins and inflammatory mediators affecting the cochlea, particularly via the round window membrane. This leads to cochlear hair cell damage and alters blood flow, worsening hearing function.^10^ Toxins produced during the chronic otitis media infection can cross the round window membrane, leading to alterations in its permeability that may damage the hair cells in the basal turn of the cochlea. Additionally, chronic inflammation of the round window can disrupt circulatory processes through intermittent vasodilation and vasoconstriction of the vessels, negatively impacting the middle ear. These toxins also pose a risk to the organ of Corti.^11^ Diagnosis relies on pure-tone audiometry (PTA) for hearing threshold assessment, with tympanometry, otoacoustic emissions, and auditory brainstem response aiding in evaluation.^12^ Treatment focuses on infection control and hearing restoration. Topical fluoroquinolones (e.g., ciprofloxacin) are preferred over aminoglycosides due to ototoxicity risks. Tympanoplasty is commonly performed to repair TM perforations, while mastoidectomy may be necessary for extensive disease.^13^ The extent and pattern of SNHL in patients with tubotympanic type CSOM remain under-investigated, and scarce research has comprehensively evaluated this association, which emphasizes the need for the current study.

This study aimed to investigate the frequency of SNHL in tubotympanic CSOM and to determine its audiological profile based on a single-center study population, providing insights for early diagnosis, intervention, and prevention of permanent hearing impairment.

## 2. METHODS

This cross-sectional descriptive study was conducted at the Otolaryngology and Audiovestibular Consultation Unit, an academic medical center, from September 10, 2024, to January 10, 2025. Using the consecutive sampling method, a total of 60 patients were enrolled. Inclusion criteria included: patients with unilateral tubotympanic CSOM with the opposite ear serving as a control. Exclusion criteria included: Bilateral CSOM, patients over 45 years old, history of noise exposure, prior ear surgery, head trauma, ototoxic medication use, chronic illnesses, pre-existing SNHL in the other ear, and patients with positive family history of congenital or acquired hearing loss. Exclusion criteria aimed to minimize confounders as much as possible.

This study was approved by the ethics committee of the Otolaryngology and Audiovestibular Consultation Unit/College of Medicine/Mustansiriyah University according to document number 8041 in 8/10/2024, ensuring adherence to the Declaration of Helsinki and local regulatory guidelines. Informed consent was obtained from all participants after explaining the study objectives, procedures, risks, and benefits, with assurances of voluntary participation, confidentiality, and the right to withdraw at any time. Special care was taken to monitor and manage any patient discomfort during procedures such as suctioning and audiometry procedures, ensuring patient safety and well-being.

Procedure after the verification of the diagnosis of tubotympanic type CSOM was made by an Ear, Nose, and Throat (ENT) specialist, an extensive history was taken from all participants, and ear examination using otoscopy and 0° rigid 50 mm endoscopy was performed to assess TM perforation size and site. Perforation size was classified as follows: small if one quadrant was involved, medium if two quadrants were involved, large if three or more quadrants were involved, and subtotal when all quadrants were involved with the remaining tympanic rim. Perforation site was classified with respect to the handle of the malleus into anterior, posterior, mallear if the handle was visualized, and big central if all quadrants were involved. Tuning fork tests (Rinne and Weber, using 256 and 512 Hz forks) were conducted.

Audiological assessment included tympanometry using the Amplivox Otowave 102 Tympanometer (Amplivox Ltd., UK) for the non-diseased ear and PTA in a soundproof booth that conformed to the maximum permissible ambient noise levels set by the American National Standards Institute (ANSI/ASA) and the International Organization for Standardization (ISO) using the calibrated Diagnostic Audiometer (Amplivox Model 240 Ltd., UK). Air conduction thresholds were established at 250, 500, 1000, 2000, 4000, and 8000 Hz, and bone conduction thresholds at 500, 1000, 2000, and 4000 Hz using the modified Hughson-Westlake procedure, with narrow-band noise masking applied when needed. Hearing loss classification was based on air-bone gap criteria: ≥10 dB for conductive or mixed loss and <10 dB for SNHL.

### 2.1 Data analysis

The primary outcome of this study was to investigate the frequency of SNHL in patients with tubotympanic CSOM. SNHL was defined as an average bone conduction threshold of more than 25 dB across the four tested frequencies (500, 1000, 2000, and 4000 Hz) using pure tone audiometry. The secondary outcomes included assessing the correlation between the occurrence of SNHL and various clinical factors such as age, gender, duration of the disease, laterality, and the size and site of TM perforation. In addition, a frequency-specific analysis was performed to identify which frequencies were most affected by the sensorineural component.

SPSS version 28 (IBM Corp., Armonk, NY) was used for statistical analysis. Continuous variables were presented as mean and standard deviation, and categorical variables were presented as frequency and percentages. Non-parametric Mann-Whitney U test and Fisher’s exact test were applied because the data distribution did not show normality when tested. The chi-square test was not applicable due to the small sample size of the group with mixed hearing loss. P-value ≤ 0.05 was considered significant. Line-marker graphs illustrated hearing loss patterns.

## 3. RESULTS

A total of 60 patients were recruited. Females contributed 53.3% (32 patients), and males for 46.7% (28 patients). Mean age was 30.6 ± 8.7 SD years. Disease duration varied: 25% (15 patients) had the condition for less than 5 years, 48.3% (29 patients) for 5 to 10 years, and 26.7% (16 patients) for more than 10 years (mean: 8.27 ± 4.1 SD years). The most common presenting clinical feature was hearing loss in 86.7% (51 patients), followed by ear discharge in 45.0% (26 patients), tinnitus in 36.7% (22 patients), and dizziness in 13.3% (8 patients), as shown in Table 1.

In this study, the left ear was affected in 58.3% (35 patients) and the right ear in 41.7% (25 patients). TM perforation characteristics were shown in Table 2, where small perforations were the most common (30.0%), and the most common perforation site was posterior in 33.3% (20 patients).

Small when one quadrant was involved, medium when two quadrants were involved, large when three or more quadrants were involved, and subtotal when all quadrants were involved with the remaining tympanic rim.

In the current study, CHL appeared to be the most prevalent, affecting 51 patients (85%), while nine patients (15%) showed mixed hearing loss.

A significant correlation was identified between the duration of the disease and the development of SNHL in this study (P = 0.018), while the age, gender, and affected side showed no significant association with the SNHL (P > 0.05), as shown in Table 3.

**Figure 1:**
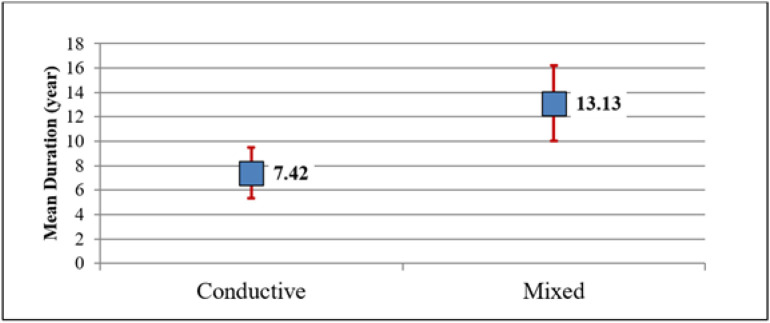
Mean disease duration in patients with conductive hearing loss versus patients with mixed hearing loss.

Figure 1 illustrates the significantly longer mean disease duration in patients with mixed hearing loss, 13.13 years, compared to those with CHL, 7.42 years, P = 0.003.

The current study revealed a statistically significant association between the SNHL and the size of the perforation, while the perforation site showed no significant association, as shown in Table 4.

Bone conduction thresholds were significantly more elevated at 500, 1000, 2000, and 4000 Hz in the diseased ear than in the control ear in patients with mixed hearing loss (P < 0.001; Table 5).

## 4. DISCUSSION

This study highlighted the frequency and characteristics of SNHL in the tubotympanic variety of CSOM using PTA. TM perforation, ossicular chain damage, and discontinuity within this intricate structure can result in significant CHL, ranging from 15 to 60 dB. Crucially, the incus-especially its long process, frequently exhibits necrosis in patients with chronic otitis media.^14^ Moreover, incomplete ossicular discontinuity has been associated with high-frequency CHL, which can serve as a useful diagnostic indicator in non-cholesteatomous CSOM.^15^ This underscores the importance of understanding and addressing these conditions to improve patient outcomes. While CSOM primarily causes CHL, SNHL can occur due to the anatomical proximity of the inner and middle ear, allowing inflammatory mediators and toxins to permeate through the round window membrane, leading to cochlear damage.^16^ The frequency of Mixed hearing loss in this study was 15%. This is consistent with previous studies by George,^17^ and Pramanik et al.,^18^ which demonstrated significant SNHL occurrence in tubotympanic CSOM due to prolonged disease activity and inflammatory mediators. Babu also reported a 10% incidence of SNHL in similar cases, which further supports this association.^19^ In a study done by Singer et al., mixed hearing loss was determined in 20 out of 200 patients (10%) with tubotympanic CSOM, emphasizing the impact of chronic inflammation on cochlear function.^20^ Disease duration was an important factor in the current study, with significantly higher SNHL prevalence in patients with a history exceeding ten years (P = 0.018). This aligns with findings by Babu and Soni et al., linking prolonged CSOM to irreversible cochlear damage.^19,21^ Prolonged disease exposure is believed to contribute to progressive cochlear damage due to chronic inflammation and toxin permeation through the round window membrane, as previously reported by Singer et al. and Abdul-Aziz et al.^20,22^ TM perforation size, rather than its anatomical location, showed a stronger correlation with SNHL in the current study. Larger perforations were more frequently associated with mixed hearing loss (P = 0.036). This aligns with studies by Soni et al. and Thakur et al., which found greater cochlear involvement in patients with subtotal and large perforations.^21,23^ Clinical evidence from Philipose et al and Tang et al. suggested that larger perforations increase exposure to inflammatory and bacterial toxins in addition to noise, potentially accelerating cochlear damage.^24,25^ In this study, age and gender did not show a significant correlation with SNHL. Although mixed hearing loss was more prevalent in older age groups, statistical analysis did not confirm a significant association (P = 0.204). Similarly, gender distribution was balanced with no statistically significant association (P = 0.721). This is consistent with prior studies by Soni et al. and Rana et al.,^21,26^ reinforcing that SNHL development in CSOM is influenced more by disease chronicity than by patient demographics, as identified by Singer et al. in their study.^20^ In this study, no significant association was determined between SNHL and ear side (P = 0.722), which is in agreement with findings of Rana et al., who reported that laterality does not impact SNHL development.^26^ The current study showed that bone conduction thresholds were significantly elevated at 500, 1000, 2000, and 4000 Hz in the affected ear (P < 0.001), supporting the cochlear involvement hypothesis in CSOM. The highest mean bone conduction threshold was observed at 4000 Hz, followed by 2000, 1000, and 500 Hz. This aligns with findings by Philipose et al. and Tang et al., who reported increased bone conduction thresholds in chronic otitis media patients, particularly at higher frequencies, due to cochlear vulnerability to chronic inflammation and toxin permeation through the round window membrane.^24,25^ The significant elevation in bone conduction thresholds in affected ears underscores the need for early tympanoplasty to mitigate SNHL progression and reduce reliance on hearing aids. These findings emphasize the importance of timely management in CSOM cases to preserve auditory function and prevent irreversible cochlear damage.

### 4.1 Limitations of this study

The cross-sectional nature of the study design limited the ability to follow the progression of SNHL. The relatively small sample size, absence of prior sample size calculation, and single-center design may limit the generalizability of the findings and reduce the statistical power to detect subtle effects. Additionally, reliance on patient-reported information increases the potential for recall bias.

## 5. CONCLUSION

CSOM, especially the tubotympanic type, is a major cause of hearing impairment. This study found a significant relation between SNHL and tubotympanic CSOM, linked to longer disease duration and larger perforations. Bone conduction thresholds were significantly higher in the affected ear, with high frequencies more impacted than speech frequencies. Periodic monitoring of patients’ hearing status, early intervention, and proper management are recommended to prevent disabling hearing impairment and improve patient outcomes.

For future research, longitudinal studies like a cohort with a larger, systematically calculated sample size, and multicenter studies are recommended to confirm these results.

## Figures and Tables

**Table 1.** Demographics, disease duration, and presenting clinical features of the study participants.

Variable		No.	%
Age, years	15–25	16	26.7
	26–35	27	45.0
	36–45	17	28.3
	Mean (SD)	30.6 (8.7)	
Gender	Male	28	46.7
	Female	32	53.3
Duration	<5 years	15	25.0
	5–10 years	29	48.3
	>10 years	16	26.7
	Mean (SD)	8.27 (4.1)	
Clinical feature	Hearing loss	52	86.7
	Ear discharge	27	45.0
	Tinnitus	22	36.7
	Dizziness	8	13.3

**Table 2.** Ear involvement, tympanic membrane perforation size, and the site of the study participants.

Variable		No.	%
Side/ear	Left	35	58.3
	Right	25	41.7
Size of perforation	Small	18	30.0
	Large	16	26.7
	Medium	13	21.7
	Subtotal	13	21.7
Site of perforation	Posterior	20	33.3
	Anterior	15	25.0
	Big central	13	21.7
	Mallear	12	20.0

**Table 3.** Relation of study participants’ hearing loss type with disease duration, age, gender, and ear side.

Variable		Conductive		Mixed		Total		*P* value
		No.	%	No.	%	No.	%	
Disease duration	<5 years	14	27.5	1	11.1	15	25.5	0.018
	5–10 years	27	52.9	2	22.2	29	48.3	
	>10 years	10	19.6	6	66.7	16	26.7	
Age in years	15–25	15	29.4	1	11.1	16	26.7	0.204
	26–35	24	47.1	3	33.3	27	45.0	
	36–45	12	23.5	5	55.6	17	28.3	
Gender	Male	23	45.1	5	55.6	28	46.7	0.721
	Female	28	54.9	4	44.4	32	53.3	
Side	Right ear	22	43.1	3	33.3	25	41.7	0.722
	Left ear	29	56.9	6	66.7	35	58.3	

Fisher’s exact test is used for comparisons.

**Table 4.** Relation of tympanic membrane perforation size and site with patients’ hearing loss type.

Variables		Conductive		Mixed		Total		*P* value
		No.	%	No.	%	No.	%	
Size of perforation	Small	18	35.3	0	0.0	18	30.0	**0.036**
	Medium	12	23.5	1	11.1	13	21.7	
	Large	12	23.5	4	44.4	16	26.7	
	Subtotal	9	17.6	4	44.4	13	21.7	
Site of perforation	Anterior	16	31.4	1	11.1	17	28.3	0.328
	Posterior	18	35.3	3	33.3	21	35.0	
	Subtotal	9	17.6	4	44.4	13	21.7	
	Mallear	8	15.7	1	11.1	9	15.0	

Fisher’s exact test is used for comparison. Bold values indicate statistical significance at *p* ≤ 0.05.

**Table 5.** Comparison of mean bone conduction thresholds in the diseased ear versus the control ear of the patients with mixed hearing loss.

Frequency (Hz)	Bone conduction threshold (dB)				*P* value
	Diseased ear		Control ear		
	Mean	SD	Mean	SD	
500	28.3	5.6	11.1	2.2	**<0.001**
1000	37.2	7.9	10.0	2.5	**<0.001**
2000	47.2	6.2	10.0	0.0	**<0.001**
4000	61.1	10.5	7.2	3.6	**<0.001**
Overall	43.5	14.1	9.6	1.7	**<0.001**

The Mann-Whitney *U* test was used for comparison. Bold values indicate statistical significance at *p* ≤ 0.05.
